# Pep2TCR: Accurate prediction of CD4 T cell receptor binding specificity through transfer learning and ensemble approach

**DOI:** 10.1002/imo2.43

**Published:** 2024-11-09

**Authors:** Kaixuan Diao, Tao Wu, Xiangyu Zhao, Nan Wang, Die Qiu, Wei‐Liang Wang, Xinxiang Li, Xue‐Song Liu

**Affiliations:** ^1^ School of Life Science and Technology ShanghaiTech University Shanghai China; ^2^ Shanghai Institute of Biochemistry and Cell Biology Chinese Academy of Sciences Shanghai China; ^3^ University of Chinese Academy of Sciences Beijing China; ^4^ Department of Dermatology Yangjiang People's Hospital affiliated to Guangdong Medical University Yangjiang Guangdong China; ^5^ Department of Colorectal Surgery Fudan University Shanghai Cancer Center Xuhui Shanghai China; ^6^ School of Life Science and Technology Shanghai Clinical Research and Trial Center ShanghaiTech University Shanghai China

## Abstract

Pep2TCR is an advanced deep learning model designed to predict cluster of differentiation 4 (CD4) T cell receptor (TCR) binding specificity, addressing the challenge posed by limited CD4 TCR data. It shows marked improvement over existing models. Pep2TCR is accessible via a user‐friendly website for predicting CD4 TCR specificity at http://pep2tcr.liuxslab.com. This innovative tool holds promise for advancing personalized cancer immunotherapies.

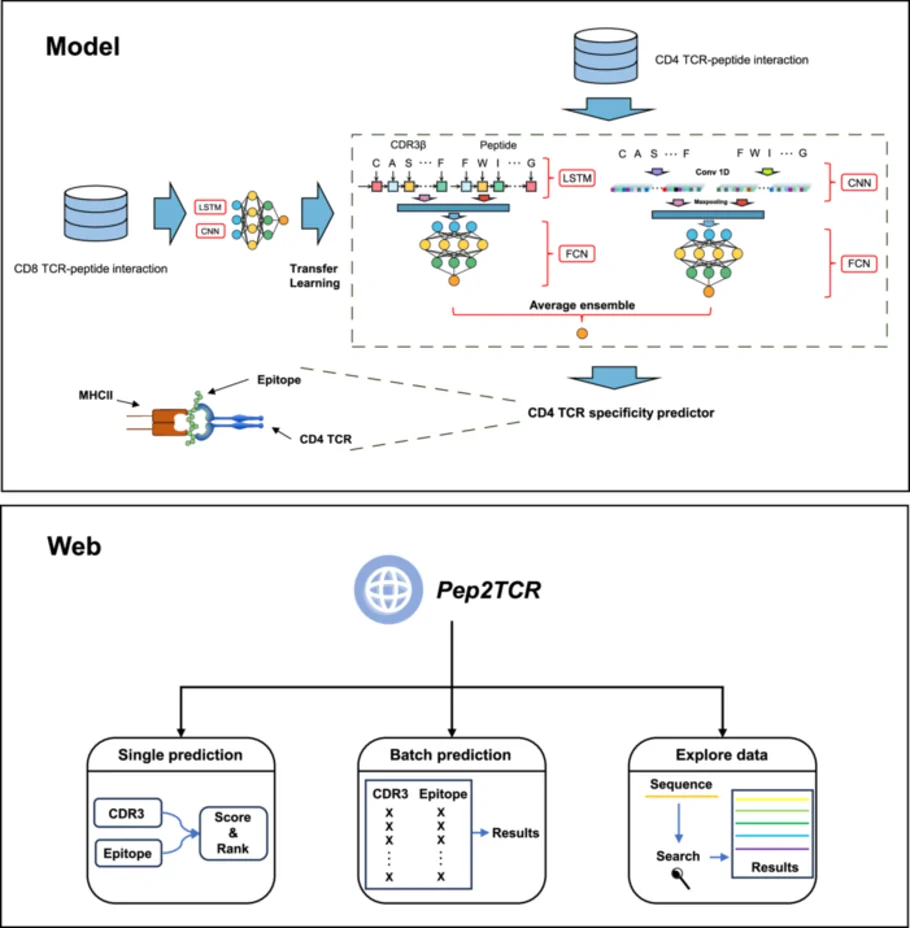

Neoantigens are short peptides ranging from 8 to 30 amino acids that are presented on the cell surface by the major histocompatibility complex (MHC). These peptides are derived from mutated DNA within tumor cells and are crucial in immunoediting [1]. MHC molecules present these neoantigens, forming peptide–MHC (pMHC) complexes, which then bind to T cell receptors (TCRs) on T cell surfaces, triggering targeted immune responses [2]. While previous emphasis in tumor immunotherapy focused on CD8^+^ T cells, the role of CD4^+^ T cells was often overlooked. However, practical immune therapy has revealed that relying solely on activating CD8^+^ T cells falls short in tumor elimination; the combined efforts of CD4^+^ and CD8^+^ T cells are necessary [3]. CD4^+^ T cells exhibit diverse functions within tumors, secreting cytokines like IL‐2, IFNγ, and TNF to sustain antitumor activity [4]. Furthermore, existing reports have indicated that neoantigen‐reactive CD4^+^ T cells hold tremendous potential for enhancing immunotherapy [5]. Therefore, focused exploration of CD4^+^ T cell TCR specificity is of paramount importance, offering deeper insights and potential for cancer immunotherapy.

However, there is limited knowledge regarding the binding specificity between the neoantigens and their corresponding TCRs. Various experimental techniques have been developed to study the binding between TCRs and pMHCs, including tetramer analysis and TCR sequencing‐based tetramers [6, 7]. However, these methods are time‐consuming, labor‐intensive, and expensive. The accumulation of TCR‐related sequencing data has led to the emergence of several comprehensive TCR‐related databases [8, 9]. Given the extensive data available, there is an urgent need to utilize bioinformatics methods for exploration. In 2020, Springer et al. developed pEptide tcR matchinG predictiOn (ERGO) [10], which utilized ERGO‐LSTM and ERGO‐AE networks constructed with long short‐term memory (LSTM) and autoencoder (AE) to predict TCR specificity. In 2021, Xu et al. proposed DLpTCR [11], a new deep‐learning model that employed an ensemble strategy to predict cluster of differentiation 8 (CD8) TCR specificity. In 2023, Pham et al. proposed epiTCR [12], a random forest‐based method that achieved good performance in predicting CD8 TCR–peptide binding specificity. Other related studies include TCRAI, Panpep (Pan‐Peptide Meta Learning), and pMTnet (pMHC–TCR binding prediction network), among others [13, 14, 15]. However, these existing tools primarily focus on CD8 TCRs and lack effectiveness in predicting CD4 TCR specificity due to limited CD4 data. Given the importance of CD4^+^ T cells, there is an urgent need for accurate prediction of CD4 TCR specificity.

In this study, we developed an effective model called Pep2TCR to address this issue (Figure 1A). We created CD8 TCR specificity prediction models (referred to as CD8 models, including LSTM and convolutional neural network [CNN] models, see Supporting Information Methods) and CD4 TCR specificity prediction models (referred to as CD4 models) by gathering TCR–peptide binding data from public databases and literature sources [8, 9, 11, 14, 15]. Due to the abundance of CD8‐related data compared to limited CD4‐related data, we employed a transfer learning strategy. This allowed us to transfer the parameters from the CD8 models to the CD4 models. We further optimized the transferred CD4 models using an ensemble approach, resulting in accurate predictions for CD4 TCR specificity. By incorporating information on tumor neoantigens and the prediction outcomes of Pep2TCR [16, 17], we observed that CD4^+^ T cells activated by MHC II neoantigens exhibited higher cytotoxicity, increased clonal frequency, and a tendency to be in an exhausted state. To summarize, Pep2TCR is a valuable tool for predicting CD4 TCR specificity and has applications in the field of immunology.

**Figure 1:**
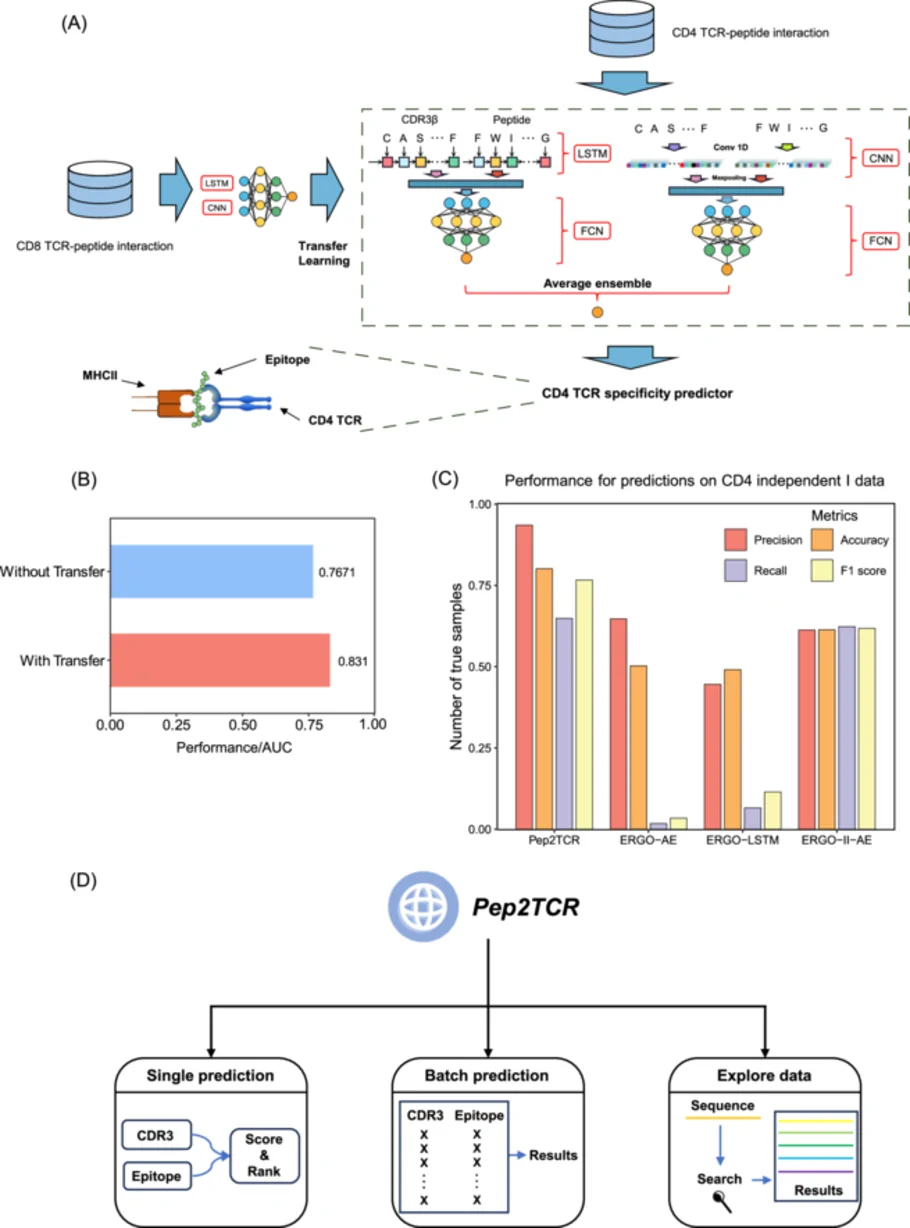
The Pep2TCR model for CD4 TCR specificity prediction. (A) The architecture of the Pep2TCR model, please note that Pep2TCR does not include MHC information as input due to the lack of comprehensive MHC data in training data sets. (B) ROC–AUC values of Pep2TCR on the combined CD4 independent validation data with and without transfer learning. (C) Precision, recall, *f*1 score, and accuracy of Pep2TCR, ERGO, and ERGOII on the CD4 independent validation data set I. (D) A flowchart of the Pep2TCR website. The “Single mode” page provides prediction for a pair of CDR3–peptide. The “Batch mode” page allows users to provide a CSV file for bulk prediction. The “Search page” allows users to find samples within a certain edit distance from the collected CD4 data. AUC, area under the curve; CD4, cluster of differentiation 4; CDR, complementarity determining region; CNN, convolutional neural network; ERGO, pEptide tcR matchinG predictiOn; FCN, fully convolutional network; LSTM, long short‐term memory; MHC, major histocompatibility complex; ROC, receiver operating characteristic; TCR, T cell receptor.

## 1 TRANSFER LEARNING AND ENSEMBLE APPROACH IMPROVE THE PERFORMANCE OF CD4 TCR SPECIFICITY PREDICTION MODELS

Our analysis reveals that the CDR3β length distribution of CD8 TCRs matches that of CD4 TCRs, despite differences in the typical length of their respective binding epitopes, with CD8 TCRs binding around nine amino acids and CD4 TCRs binding around 15 amino acids (Figure S2A,B). Given that the volume of CD8 TCR specificity data is nearly nine times that of CD4 TCR specificity data, we utilized a transfer learning strategy to pretrain models for the downstream task (CD4 TCR specificity prediction). Initially, we trained and evaluated CD8 models. In Figure S3A, the CD8 LSTM and CNN models demonstrated good receiver operating characteristic–area under the curve (ROC–AUC) and precision‐recall–area under the curve (PR–AUC). Subsequently, we selected the best‐performing models from the 10‐fold cross‐validation as our final CD8 models. On the CD8 independent validation data set, both the CD8 LSTM and CD8 CNN models demonstrated good generalization ability (Figure S3B). For detailed data processing and negative sampling methods, please refer to the Supporting Information Methods and Figure S1.

We started by transferring the parameters of the CD8 models to the CD4 models to initialize the parameter set effectively. On the CD4 independent validation data set I, we observed a significant performance enhancement for the CD4 CNN model with transfer learning (Figure S4A,B). These results suggest that the effects of transfer learning vary depending on the model type. To further enhance the performance of CD4 models, we used the ensemble approach. We constructed two models: Avg‐Ensemble and Sub‐Ensemble (see Supporting Information Methods). The analysis results demonstrate that Avg‐Ensemble displayed significant improvement on the CD4 independent validation data set I, suggesting improved generalization ability (Figure S4C,D). As a result, we selected Avg‐Ensemble as the final model named “Pep2TCR.” Furthermore, Figure 1B illustrates a significant improvement in Pep2TCR after applying transfer learning.

Additionally, we sought to evaluate the performance of a large language model. Given the relatively small size of our training data set, we selected the ESM2_t6_8M_UR50D version based on its balance of performance and computational efficiency [18]. We employed ESM2 as a feature extractor for TCR and antigen peptides. The extracted features were then fed into the MLP to predict TCR–peptide binding interactions (Method). Figure S5A,B indicated that the ESM2‐based model achieved performance comparable to our transfer learning‐based CNN model. However, our transferred LSTM still outperformed the ESM2 model for this specific task. This outcome suggests that pretraining on the CD8 data set provides advantages over directly applying large‐scale, general protein language models for predicting TCR–peptide interactions.

## 2 PEP2TCR OUTPERFORMS EXISTING METHODS IN CD4 TCR SPECIFICITY PREDICTION

We compared the performance of Pep2TCR with existing CD4 TCR specificity prediction tools using two independent CD4 TCR specificity data sets. First, we compared Pep2TCR with conventional classifiers. The results are shown in Figure S4E,F, indicating that Pep2TCR has shown a clear superiority over these conventional classifiers. Next, we compared Pep2TCR with the ERGO series models [10, 19]. Pep2TCR achieved greater ROC–AUCs and PR–AUCs on independent validation data sets I and II (Figures S6A,B and S7A,B). In other metrics, Pep2TCR also outperforms ERGO on independent validation data set I (Figures 1C and S7C) and independent validation data set II (Figures S6C and S7D). Detailed confusion matrices also demonstrate the strong generalization ability of Pep2TCR (Figure S8A). In addition, we show ROC–AUCs of Pep2TCR and ERGO on four most common epitopes in the combined CD4 validation data set (Figure S8B). The performance of Pep2TCR on the CD8 data set is shown on Figure S7E,F, and its performance remains stable across CD4 independent data sets with varied sequence similarity thresholds (Figure S8C,D, detailed in Supporting Information Results). Therefore, Pep2TCR shows significantly improved performance compared with existing tools in predicting the binding specificity of CD4 TCR–peptide.

we also provide a binding rank value, where smaller values indicate stronger binding. We randomly selected 1000 CD4 TCRs as a background and defined two thresholds: a rank value within 0.05 represents strong binding, while within 0.1 represents weak binding. We used the combined CD4 independent validation data set to evaluate model performance. It is evident that all metrics of Pep2TCR significantly surpass the metrics of ERGO, demonstrating the improved generalization ability of Pep2TCR compared with existing tools (Figure S9).

## 3 PEP2TCR HAS GOOD PERFORMANCE ON UNSEEN PEPTIDE PREDICTION

To evaluate how well Pep2TCR can predict TCR recognition of peptides that were not included in the training data set (referred to as “unseen peptides”), we partitioned the positive pairs of two CD4 independent validation data sets into a shared peptide data set and a unique peptide data set. We used the shuffle method to balance them (Supporting Information Methods). The share peptide data set consists 158 peptide–TCR pairs and 12 peptides that were already included in the training data, while the unique peptide data set consists 1527 peptide–TCR pairs and 34 peptides that had never been seen in the training data. Figure S6D and S6E illustrate the performance of Pep2TCR and ERGO on these two data sets. The results demonstrate that Pep2TCR outperforms existing tools on both data sets. The TCR prediction for unseen peptide is still a significant scientific challenge [14, 20]. Our results suggest that Pep2TCR has strong generalization capabilities when it comes to predicting TCR recognition of unseen peptides.

## 4 APPLICATION OF PEP2TCR FOR IDENTIFYING NEOANTIGEN‐REACTIVE CD4^+^ T CELL SIGNATURES

We analyzed the single‐cell data set of infiltrating CD4^+^ T cells in gastrointestinal tumors from Zheng et al.'s study [16]. This data set contains transcriptome data, TCR sequencing data, and genomic DNA mutation status of patients. Our objective was to identify signatures of CD4^+^ T cells activated by neoantigens. To achieve this, we utilized the mutation pool mainly mentioned in the study (called PP1 pool, a series of point mutations) along with netMHCIIpan 4.0 [17] to predict MHCII‐presented neoantigens, setting thresholds below 5% for binding neoantigens. We obtained numerous CD4 TCRs from the TCR sequencing data, and subsequently used Pep2TCR to predict the binding between CD4 TCRs and predicted neoantigens, aiming to identify neoantigen‐reactive CD4^+^ T cells with a prediction score threshold of 0.54 to control the number of neoantigen‐reactive CD4^+^ T cells. In total, we identified 479 neoantigen‐reactive CD4^+^ T cells and 714 CD4^+^ T cells that were not activated by neoantigens. While the majority of CD4^+^ T cells do not typically exhibit cytotoxic functions, it is important to note that under certain conditions, such as in response to neoantigens, CD4^+^ T cells can acquire cytotoxic capabilities. We calculated normalized exhaustion and cytotoxicity scores (see Supporting Information Methods for details and limitations), which revealed that neoantigen‐reactive CD4^+^ T cells displayed higher exhaustion and cytotoxicity (Figure S10A). Moreover, these cells exhibited elevated clonal levels (Figure S10B). This is consistent with Zheng et al.'s study. We also calculated the proportions of cells expressing high levels of *IL7R*, *HOPX*, or *ADGRG1*. This analysis indicated enhanced expression of the effector/memory Th1 cell marker *HOPX* and the cytotoxicity‐related gene *ADGRG1*, while the memory T‐cell marker *IL7R* expression was reduced in neoantigen‐reactive CD4^+^ T cells (Figure S10C), suggesting that neoantigen‐reactive CD4^+^ T cells are likely cytotoxic effector Th1 cells. These findings are in line with Zheng's study [16]. In summary, neoantigen‐reactive CD4^+^ T cells demonstrate potent cytotoxicity and undergo clonal expansion for effective tumor cell elimination. However, excessive responses can lead to cellular exhaustion.

## 5 DISCUSSION

The precise identification and prediction of TCR–peptide binding have significant implications in various immunological domains, such as vaccine and TCR‐T design. In this study, we have developed advanced Pep2TCR to predict CD4 TCR–peptide interactions by utilizing transfer learning and ensemble learning techniques, given the critical role of CD4^+^ T cells in immunity and the absence of suitable predictive tools. In the context of TCR specificity prediction, our study has harnessed transfer learning to bridge the data gap between CD8 and CD4 T cell models. Notably, our investigation has revealed that the utility of transfer learning is influenced by the architectural characteristics of the models employed.

The transfer learning approach employed in Pep2TCR played a pivotal role in bridging the data gap between CD8 and CD4 T cell models, ultimately enabling accurate predictions of CD4 TCR–peptide interactions. By leveraging the knowledge and representations learned from the abundant CD8 T cell data, our models could effectively generalize to the CD4 domain, where data scarcity has traditionally posed a significant challenge. In such cases, the transferred knowledge from the CD8 domain acted as a crucial prior, enabling the models to make more accurate predictions and generalize better to unseen CD4 contexts (Figures 1B, S4, and S5). This finding underscores the potential of transfer learning as a powerful tool for overcoming data scarcity challenges in immunological and biological applications.

Pep2TCR surpassed other established and conventional models, with extensive applications in exploring immunity, such as validating signatures of neoantigen‐reactive CD4^+^ T cells. Those results validated the effectiveness of transfer learning and ensemble learning techniques. One interesting observation is that in our CD4 data sets (Figure S2C), the epitope source distribution differs. The epitope source in both CD4 independent data sets is mainly derived from various cancers, while the epitope source in the CD4 training data set mainly focuses on pathogens. This difference makes the independent data sets more independent and validates the performance of Pep2TCR once again.

It is important to note that Pep2TCR currently focuses only on the CDR3 region of TCR β‐chains and peptide information, while disregarding α‐chains and MHC details. This aligns with prevailing approaches due to limited data availability and the relatively lower significance of MHC information in this context (Supporting Information Methods). However, as more data becomes available, it could be beneficial to incorporate MHC and α‐chain data into Pep2TCR for improved predictions. Although Pep2TCR outperforms current models, there is still room for enhancement. Possible avenues for improvement include: (1) Expanding the training data set to enhance generalization capability; (2) Considering the integration of MHC and α‐chain data, especially with larger data set sizes, to achieve more accurate predictions; (3) Currently, Pep2TCR does not account for ternary complex structural information due to the lack of 3D crystal structure data. We anticipate that incorporating structural details will provide a new perspective for model learning and enhance Pep2TCR's performance.

Finally, researchers can utilize Pep2TCR by accessing our Docker image at https://hub.docker.com/r/liuxslab/pep2tcr and website at http://pep2tcr.liuxslab.com. With the emergence of precision medicine, we anticipate that Pep2TCR has the potential to propel tumor immunology research forward and offer valuable support to immunotherapies.

## 6 CONCLUSION

Pep2TCR is an innovative model that accurately predicts CD4 TCR binding specificity using transfer learning and ensemble approaches. By leveraging abundant CD8 TCR data, it effectively bridges the data gap in CD4 TCR specificity prediction. Pep2TCR demonstrates superior performance compared to existing methods and holds significant promise for advancing personalized cancer immunotherapies.

## AUTHOR CONTRIBUTIONS

**Kaixuan Diao**: Writing—original draft; writing—review and editing; visualization; validation; methodology; software; formal analysis; project administration; data curation. **Tao Wu**: Investigation; writing—review and editing. **Xiangyu Zhao**: Data curation. **Nan Wang**: Data curation. **Die Qiu**: Data curation. **Wei‐Liang Wang**: Supervision. **Xinxiang Li**: Supervision; writing—review and editing; project administration. **Xue‐Song Liu**: Writing—review and editing; conceptualization; data curation; supervision; funding acquisition; resources; project administration.

## CONFLICT OF INTEREST STATEMENT

The authors declare no conflict of interest.

## ETHICS STATEMENT

No animals or humans were involved in this study.

## Supporting information

Figure S1 Data processing workflow.
Figure S2 Distribution of independent validation data for CD8 and CD4 models.
Figure S3 The trained CD8 models demonstrate good generalization ability.
Figure S4 Transfer learning and ensemble approach enhance the performance of TCR specificity prediction models.
Figure S5 Pep2TCR outperforms ESM2‐based model.
Figure S6 Pep2TCR surpasses existing tools in CD4 TCR specificity prediction.
Figure S7 Pep2TCR outperforms existing tools in CD4 TCR specificity prediction.
Figure S8 Validating the performance of Pep2TCR.
Figure S9 Validation of Pep2TCR in the context of binding rank.
Figure S10 Application of Pep2TCR in recognizing the signatures of neoantigen‐reactive CD4^+^ T cell.

## Data Availability

The data that support the findings of this study are openly available in Pep2TCR at https://github.com/XSLiuLab/Pep2TCR. The Pep2TCR package developed in the study is available on GitHub at https://github.com/XSLiuLab/Pep2TCR. The collected data are on GitHub at https://github.com/XSLiuLab/Pep2TCR/tree/main/data. Pep2TCR docker is on DockerHub at https://hub.docker.com/r/liuxslab/pep2tcr. Pep2TCR website is available at http://pep2tcr.liuxslab.com. Supporting Information Materials (figures, scripts, graphical abstract, slides, videos, Chinese translated version and update materials) may be found in the online DOI or iMeta Science http://www.imeta.science/imetaomics/.
