# Supplementary material for: Pep2TCR: Accurate prediction of CD4 T cell receptor binding specificity through transfer learning and ensemble approach
Source: IMetaOmics. 2024 Nov 9;1(2):e43. doi: 10.1002/imo2.43 (PMC12806441; doi:10.1002/imo2.43)
Supplement: Supplementary file 1 — Figure S1 Data processing workflow. Figure S2 Distribution of independent validation data for CD8 and CD4 models. Figure S3 The trained CD8 models demonstrate good generalization ability. Figure S4 Transfer learning and ensemble approach enhance the performance of TCR specificity prediction models. Figure S5 Pep2TCR outperforms ESM2‐based model. Figure S6 Pep2TCR surpasses existing tools in CD4 TCR specificity prediction. Figure S7 Pep2TCR outperforms existing tools in CD4 TCR specificity prediction. Figure S8 Validating the performance of Pep2TCR. Figure S9 Validation of Pep2TCR in the context of binding rank. Figure S10 Application of Pep2TCR in recognizing the signatures of neoantigen‐reactive CD4+ T cell. [file IMO2-1-e43-s001.docx]

# Supporting information to

# Pep2TCR: accurate prediction of CD4 T cell receptor binding specificity through transfer learning and ensemble approach

## Running title: Transfer Learning for CD4 TCR Binding

Kaixuan Diao^1,2,3#^, Tao Wu^1^, Xiangyu Zhao^1^, Nan Wang^1^, Die Qiu^1^, Wei-Liang Wang^4^, Xinxiang Li^5^, Xue-Song Liu^6*^

^1^ School of Life Science and Technology, ShanghaiTech University, Shanghai 201203, China;

^2^ Shanghai Institute of Biochemistry and Cell Biology, Chinese Academy of Sciences, Shanghai 200031, China;

^3^ University of Chinese Academy of Sciences, Beijing 101408, China;

^4^ Department of Dermatology, Yangjiang People's Hospital affiliated to Guangdong Medical University, Yangjiang, Guangdong 529500, China;

^5^ Department of Colorectal Surgery, Fudan University Shanghai Cancer Center, 270 Dong’an Road, Xuhui, Shanghai 200032, China;

^6^ School of Life Science and Technology, ShanghaiTech University, Shanghai Clinical Research and Trial Center, Shanghai 201203, China;

* Correspondence: [liuxs@shanghaitech.edu.cn](mailto:liuxs@shanghaitech.edu.cn) (Xue-Song Liu)

## SUPPLEMENTARY RESULT

### Overview of Pep2TCR

Pep2TCR presents a novel deep learning architecture to predict CD4 TCR-peptide binding. This method is inspired by transfer learning and ensemble learning, as shown in Figure 1A. Transfer learning utilizes existing knowledge from related domains. It achieves this by transferring pre-trained model parameters to a new model, thereby improving learning ability [1,2]. On the other hand, ensemble learning combines multiple weak learners to create a strong learner, thus enhancing the final model's generalization ability, such as DLpTCR and random forests [3,4]. Given the large volume of CD8 TCR and corresponding peptide data, our goal is to leverage the strengths of transfer learning and ensemble learning to develop an effective CD4 TCR specificity prediction tool that can be widely used. Pep2TCR consists of three key stages: first, training the CD8 models; second, transferring these learners to the CD4 models; and finally, applying an ensemble strategy to improve overall performance. The models used include two variants: LSTM and CNN models, which use LSTM layer and CNN layer, respectively (Methods). Pep2TCR generates two outputs for each peptide-TCR pair: a prediction score and a percentile rank (also known as binding rank). The percentile rank is derived from NetMHCpan and pMTnet [2,5]. The prediction score is a continuous numerical output ranging from 0 to 1, representing the binding score. This score indicates the probability of binding between a given peptide and a given CD4 TCR. For any given peptide-TCR pair, a higher binding score indicates an increased likelihood of binding, suggesting a greater potential for TCR clonal expansion. The binding rank represents the percentile rank of the prediction score for the TCR and peptide interaction, compared to a background distribution of 1000 randomly sampled TCRs for the same peptide. A lower rank indicates a stronger binding. In this study, we collected CD8 and CD4 data from public research and databases, and generated negative samples to train and evaluate the performance of Pep2TCR (Methods).

We analyzed the distribution of data and found that the length distribution of the CDR3β region of CD8 TCRs is consistent with that of CD4 TCRs. Although CD8 TCRs and CD4 TCRs share a similar binding pattern, with their contact core region of peptides being around 9 amino acids in length, the whole length of the peptides they bind to still differs. Epitopes that bind to CD8 TCRs are typically around 9 amino acids long, whereas those binding to CD4 TCRs are generally around 15 amino acids in length (Figure S2A). In addition, the number distribution of epitopes is shown in Figure S2B. This distribution pattern is consistent with previous reports [6]. The epitope source distribution is shown in Figure S2C.

### Pep2TCR outperforms existing methods in CD4 TCR specificity prediction

Given the similarity in binding patterns of CD8 TCRs and CD4 TCRs with antigenic peptides, we are interested in evaluating the performance of CD8-specific tools on CD4 datasets, also assessing Pep2TCR's performance on CD8 datasets. To this end, we evaluated the CD8 TCR specificity prediction tools Panpep, epiTCR, and DLpTCR on the combined CD4 validation dataset. The results demonstrated that Pep2TCR significantly outperformed these CD8 TCR models (Figure S7E), indicating that despite the similarities in the binding patterns of CD8 and CD4 TCRs, there are subtle differences that Pep2TCR can capture, crucial for CD4 TCR binding. Furthermore, we evaluated Pep2TCR on the independent CD8 validation set (Figure S7F). Due to differences in binding patterns and the fact that Pep2TCR was specifically developed for CD4 TCR binding specificity, Pep2TCR’s performance in CD8 TCR specificity prediction was not good.

Meanwhile, we also demonstrate the ability of Pep2TCR to distinguish between positive and negative samples. We used the shuffle method to generate negative samples (Methods), resulting in our dataset containing two peptides for each TCR CDR3β: one binding and one non-binding. In two CD4 independent validation datasets, we calculated the ratio for each pair as follows: $ratio= \frac{True preds}{False preds}$, where $True preds$ represent the prediction score of Pep2TCR for one positive example, and $False preds$ represent the prediction score for the corresponding negative example. If the majority of ratios are greater than 1, it indicates that Pep2TCR can recognize the truly binding peptides. We present the ratio distribution for the two CD4 independent validation datasets (Figure S8C). It can be observed that log2(ratio) is mostly greater than 0, demonstrating Pep2TCR's excellent ability to distinguish positive from negative samples.

In order to make the CD4 independent datasets more independent from the CD4 training dataset, we considered sequence similarity [7] (Methods). We set four thresholds at 90%, 92%, 96% and 99% to reduce CDR3 sequence similarity on the combined CD4 independent dataset, and AUCs were accessed. The Figure S8D indicated that Pep2TCR can sustain stable performance in this situation.

### Webserver for Pep2TCR

We have developed a well-designed and user-friendly website for Pep2TCR, making it easier to predict CD4 TCR-peptide binding specificity (Figure 1D). This webserver is built using R Shiny and utilizes the Pytorch and other Python packages for computation. The website offers two modes: “Single Mode” and “Batch Mode”, catering to individual and multiple predictions, respectively. Please ensure that the length of βCDR3 region to 8-20 and the length of epitope to 9-20, and only the 20 common amino acids are acceptable. We suggest utilizing a prediction score threshold of 0.5 to indicate binding, a rank threshold of 0.05 for identifying strong binding, and a rank threshold of 0.10 for weak binding. Users can adjust these thresholds based on their specific circumstances. The “Search” page is inspired by McPAS-TCR, enabling users to explore CDR3 and antigen peptides data conveniently [8], users can find similar CDR3s or epitopes based on Levenshtein distance. The “Help” page provides detailed instructions on how to use the website and offers relevant resource links, while the “About” page displays ownership information and contact details for the project. In summary, this web service allows researchers to make quick and convenient predictions, propelling advancements in the field of immunology and potentially benefiting cancer precision immunotherapy.

## SUPPLEMENTARY METHODS

### Data curation

### CD8 TCR datasets:

Due to the abundance of researches on CD8 TCR specificity prediction, several manually curated datasets of CD8 TCR-peptide interactions have been generated. For this study, we utilized three datasets from pMTnet, Panpep, and DLpTCR to construct the CD8 training dataset and CD8 independent validation dataset**,** which we refer to CD8 data.

During the data preprocessing stage, we only utilize the CDR3 region of the TCRβ chain. This decision is based on the fact that, compared to the α chain, the β chain exhibits greater diversity, and the CDR3 region of the β chain directly interacts with antigens, making it highly diverse. Moreover, datasets containing both α and β chains are scarce and insufficient to support effective deep learning. Additionally, we do not consider MHC information for two reasons: a) an existing report suggested that incorporating MHC information has minimal impact on prediction outcomes [9]; b) datasets containing both CDR3, antigen peptides, and MHC for CD4 type are limited. Given the richness of human TCR data, we retained only human pairing data. In view of binding features, we limited our selection to antigens with lengths ranging from 8 to 11 amino acids and CDR3s with lengths from 8 to 20 amino acids. The CD8 training dataset consists of 37,656 positive samples, derived through screening of pMTnet's training dataset and Panpep's base dataset [2,10], including 691 unique peptides and 34,346 unique CDR3 sequences (Figure S1A). The CD8 independent validation dataset comprises 5,299 positive samples, obtained by screening pMTnet's test dataset and all data from DLpTCR [2,3], with 261 unique peptides and 2,385 unique CDR3 sequences.

### CD4 TCR datasets:

The CD4 data (CD4 TCR-peptide interaction data) was collected from various public databases, such as IEDB, McPAS, VDJdb, TBAdb, TCR3d, and dbPepNeo2.0 [8,11–15]. The selection criteria, apart from limiting the class II peptide length between 9 and 20, were the same as those used for CD8 data filtering. For this study, we constructed a CD4 training dataset and two CD4 independent validation datasets. The CD4 training dataset contains 3978 positive samples from the IEDB and VDJdb databases. The first independent CD4 validation dataset consists of 624 positive samples selected from McPAS, TBAdb, and TCR3d databases, while the second independent CD4 validation dataset includes 219 positive samples from the dpPepNeo2.0 database.

### Generation of negative samples:

The interaction data between TCR and peptides obtained from the database are all positive samples, meaning they are all in a binding state. Therefore, it is necessary to generate negative samples (non-binding data) to balance the datasets. This will enable the deep learning model to learn the intrinsic features of the data and enhance its discriminative ability. According to previous research [6], there are two methods for generating negative samples: the shuffle method and the negative reference method (Figure S1B). In the shuffle method, each CDR3 sequence in the positive dataset is paired with a randomly sampled epitope from the positive sequence pairs' epitopes, while excluding their already known true epitope partner(s). As for the negative reference method, CDR3 sequences from a negative TRB CDR3 reference dataset are aligned with epitopes randomly selected from the positive pairs. Since previous research indicated that the negative reference method is not conducive to enhancing the model's discriminative ability, unless otherwise specified, we adopt the shuffle method to generate negative samples to balance a given dataset.

### The representation of amino acids:

According to the existing literatures [7,16], we employed two encoding strategies based on the AAindex11 matrix and the BLOSUM50 matrix. The AAindex database (<https://www.genome.jp/aaindex/>) contains over 500 amino acid indices, with each index representing a physical and chemical property of 20 common amino acids. By organizing these indices into columns, an AAindex matrix is created, where rows represent amino acids and columns represent indices. Firstly, we z-score normalized the columns of this matrix and then reduced its dimensionality to 11 dimensions using PCA to remove redundant information (after dimensionality reduction, over 99% of the variance can be explained), resulting in the AAindex11 matrix. A peptide and paired CDR3 were then padded to a maximum length of 20 and encoded into a 20 x 11 feature matrix using the AAindex11 matrix. Additionally, the BLOSUM50 matrix is a more general method, encoding each amino acid as a 20-length vector. Therefore, the padded peptide and paired CDR3 can be encoded into a 20 x 20 feature matrix using the BLOSUM50 matrix.

### Similarity scoring

To reduce data redundancy, we considered CDR3 sequence similarity in Net2TCR-2.0 [7], as similar sequences in training and test sets can aid prediction. The goal is to trim a test set to decrease similarity, so we removed points from the test set with a Levenshtein similarity above a certain threshold. The Levenshtein similarity score between two CDR3 sequences is calculated using the equation: ${Sim}_{Lev}=\frac{\max\left( \left| u \right|, \left| v \right| \right)-{Distance}_{Lev}(u, v)}{\max\left( \left| u \right|, \left| v \right| \right)}$, where u and v are two CDR3 sequences, and |·| denotes their length, The term ${Distance}_{Lev}$ represents the Levenshtein distance between u and v.

### Model architecture

### Base models:

We have employed two model architectures, one based on LSTM and the other on CNN. According to previous research [3], the LSTM-based model (referred to as the LSTM model) takes peptides and CDR3s encoded with AAindex11 as inputs. Two LSTM layers, each with a dropout rate of 0.3 and a hidden state size of 80, process the peptides and CDR3 separately. The final hidden states from the last time step of both LSTM layers are concatenated to yield a 160-dimensional vector. Subsequently, this vector passes through a fully connected layer with 80 neurons, followed by an output layer with a single neuron activated by the sigmoid function.

Similar to NetTCR-2.0 [7], the CNN-based model (referred to as the CNN model) takes peptides and CDR3s encoded with BLOSUM50 as inputs. For each peptide and CDR3, we use 5 one-dimensional convolutional layers with kernel sizes of 1, 3, 5, 7, and 9, and an output channel size of 4 to process them. Then, a max-pooling operation with an output size of 1 is applied to extract features, which are then concatenated to form a 40-dimensional vector. Afterward, this vector goes through a fully connected layer with 32 neurons, followed by an output layer with a single neuron activated by the sigmoid function.

### Ensemble learning:

Ensemble learning is a strategy that combines multiple weak learners in a certain way to form a strong learner. In this study, we investigated two ensemble strategies: Average Ensemble (Avg-Ensemble) and Sub-Ensemble (also called stacking ensemble) based on secondary learners. Avg-Ensemble involves taking a weighted sum of the predictive outputs from multiple weak learners, with equal weights assigned to each weak learner in this study. On the other hand, Sub-Ensemble treats the predictive outputs of multiple weak learners as features and feeds them into a secondary learner (in this case, a multi-layer perceptron, MLP). The MLP used in the Sub-Ensemble consists of 5 linear layers: the first layer is the input layer with 10 neurons, followed by 3 hidden layers with 32, 16, and 8 neurons, respectively. After that, a dropout layer with a dropout rate of 0.2 is applied, and finally, the output layer with 1 neuron is used, followed by a sigmoid activation function.

### Pep2TCR model:

We have developed Pep2TCR using the transfer learning concept. Here is a brief overview of the architecture: 1) We gathered CD8-related data and constructed CD8 models based on LSTM or CNN. 2) Then, we collected CD4-related data and employed a transfer learning strategy to transfer the CD8 models to the CD4 models. 3) Finally, we used an ensemble approach to integrate the CD4 LSTM models and CNN models (5 models from 5-fold cross-validation, respectively). The predictive outcome of the ensemble model represents the final prediction result.

### ESM2-based models

We utilized the ESM2_t6_8M_UR50D version of the ESM2 model [17], a state-of-the-art pre-trained protein language model, to enhance feature extraction for TCR and antigen peptides. Given the relatively small size of our training dataset, we adopted a transfer learning approach. Specifically, we froze all layers of the pre-trained ESM2 model to serve as a feature extractor, ensuring that the model's pre-learned representations of protein sequences were retained without further modification during our training process.

The extracted features from the ESM2 model were then fed into a Multi-Layer Perceptron (MLP) for the final prediction of TCR-peptide binding interactions. The MLP was carefully tuned using Bayesian optimization to determine the optimal hyperparameters, including the learning rate, dropout rate, number of neurons in each hidden layer, and batch size. This ensured that the MLP was well-regularized and capable of effectively leveraging the features provided by ESM2.

To provide a robust benchmark for comparison, we also implemented an ensemble approach with ESM2. We trained five independent ESM2-based models, each initialized with different random seeds on our dataset. The predictions from these models were aggregated using a simple averaging method, which is a common and effective ensemble strategy. This ESM2 ensemble model was then compared with our Pep2TCR model to assess performance and reliability.

### Hyperparameters optimization:

In our study, we employed a systematic and data-driven approach to determine the optimal hyperparameters for the Pep2TCR and other models. We utilized Bayesian optimization, implemented via the Hyperopt package, to conduct an extensive search over the hyperparameter space. This included parameters such as the dropout rate, the number of neurons in hidden layer, the learning rate, and the batch size. By utilizing Bayesian optimization and a thorough evaluation process, we effectively regularized the models and achieved an optimal balance between underfitting and overfitting.

Throughout the hyperparameter tuning process, we conducted more than 40 iterations. Each iteration involved training the model with a specific set of hyperparameters, evaluating its performance on the validation set, and then adjusting the hyperparameters for subsequent iterations based on the results obtained. The validation ROAUC served as the primary metric for evaluating model performance during this process, but we also monitored other metrics such as accuracy and F1-score to ensure a comprehensive assessment. The final set of hyperparameters, including the dropout rate, was selected based on the iteration that achieved the highest validation ROAUC.

### Model training:

We separately trained CD8 models and CD4 models using the CD8 training dataset and the CD4 training dataset, respectively. Each model consists of CNN and LSTM models. We utilized Bayesian optimization to select the best hyperparameter combinations and implemented early stopping with a patience of 30 epochs to prevent model overfitting. The learning rates of CD8 model and CD4 model are 0.01 and 0.02, respectively. Both models were trained for 100 epochs, with a batch size of 512 for the CD8 model and 256 for the CD4 model. We evaluated the performance of the CD8 models using a 10-fold cross-validation and the CD4 models using a 5-fold cross-validation. We then initialized the CD4 models with the parameters obtained from the well-trained CD8 models and trained them in the same manner. At last, average ensemble strategy was applied to 10 CD4 models. We used the Adam optimizer to optimize model parameters and BCE loss function to assess the quality of model predictions. BCE is defined as shown below:

$$BCE loss= -\frac{1}{N}\sum_{i=1}^{N} y_{i}\log\left( p_{i} \right)+\left( 1-y_{i} \right)log(1-p_{i})$$

Where $y_{i}$ is the label for ${pair}_{i}$, taking values 0 or 1; $p_{i}$ is the predicted value for ${pair}_{i}$, representing the probability of CDR3β and peptide binding.

### Performance evaluation:

We adopt widely accepted metrics in bioinformatics, such as ROC-AUC, PR-AUC, recall, precision, f1 score, and accuracy, to comprehensively evaluate the performance of the models on their respective independent datasets. They are defined as follows:

$$Recall= \frac{TP}{TP+FN}$$

$$Precision= \frac{TP}{TP+FP}$$

$$F1 score= 2\cdot\frac{Recall\cdot Precision}{Recall+Precision}$$

$$Accuracy= \frac{TP+TN}{TP+TN+FP+FN}$$

Where $TP$, $TN$, $FP$, and $FN$ denote true positive, true negative, false positive, and false negative, respectively.

Furthermore, top20 and top50 represent the number of actual binding samples among the top 20 or 50 samples with the highest likelihood of prediction. The larger the value, the stronger the model's ability to identify positive samples.

### The signatures of neoantigen-reactive CD4+ T cell:

In this study, we utilized Pep2TCR to validate predictions using experimental datasets from a previous gastrointestinal cancer study [18]. Initially, we focused on mutated genes from five patients' peptide pool 1 (PP1) as the primary target. Neoantigens were identified from PP1 mutations using NetMHCIIpan4.0, resulting in 62 neoantigens and 1193 available CD4^+^ T cells with CDR3 beta sequences for further analysis.

The selection of specific genes as markers for exhaustion and cytotoxicity was informed by well-established findings from previous researches [18–20]. The exhaustion score and cytotoxicity score were calculated by determining the average TPM expression z-scores of 5 exhausted markers (*CTLA4*, *HAVCR2*, *LAG3*, *PDCD1*, and *TIGIT*) and 7 cytotoxicity markers (*PRF1*, *GNLY*, *IFNG*, *GZMA*, *CST7*, *NKG7*, and *GZMB*) respectively. While z-score normalization is a widely used technique for standardizing expression data, it relies on the assumption that the data follows a normal distribution. In practice, gene expression data may deviate from this assumption, which could impact the accuracy of the comparisons. Despite this, z-score normalization effectively captures relative differences in expression, making it valuable for comparing marker levels across different cell populations.

## REFERENCES

1. Wang, Guangshuai, Tao Wu, Wei Ning, Kaixuan Diao, Xiaoqin Sun, Jinyu Wang, Chenxu Wu, et al. 2023. “TLimmuno2: Predicting MHC Class II Antigen Immunogenicity through Transfer Learning.” *Briefings in Bioinformatics* bbad116. https://doi.org/10.1093/bib/bbad116

2. Lu, Tianshi, Ze Zhang, James Zhu, Yunguan Wang, Peixin Jiang, Xue Xiao, Chantale Bernatchez, et al. 2021. “Deep Learning-Based Prediction of the T Cell Receptor–Antigen Binding Specificity.” *Nature Machine Intelligence* 3(10): 864–75. https://doi.org/10.1038/s42256-021-00383-2

3. Xu, Zhaochun, Meng Luo, Weizhong Lin, Guangfu Xue, Pingping Wang, Xiyun Jin, Chang Xu, et al. 2021. “DLpTCR: An Ensemble Deep Learning Framework for Predicting Immunogenic Peptide Recognized by T Cell Receptor.” *Briefings in Bioinformatics* 22(6): bbab335. https://doi.org/10.1093/bib/bbab335

4. Breiman, Leo. 2001. “Random Forests.” *Machine Learning* 45(1): 5–32. https://doi.org/10.1023/A:1010933404324

5. Reynisson, Birkir, Bruno Alvarez, Sinu Paul, Bjoern Peters, and Morten Nielsen. 2020. “NetMHCpan-4.1 and NetMHCIIpan-4.0: Improved Predictions of MHC Antigen Presentation by Concurrent Motif Deconvolution and Integration of MS MHC Eluted Ligand Data.” *Nucleic Acids Research* 48(W1): W449–54.

6. Moris, Pieter, Joey De Pauw, Anna Postovskaya, Sofie Gielis, Nicolas De Neuter, Wout Bittremieux, Benson Ogunjimi, et al. 2021. “Current Challenges for Unseen-Epitope TCR Interaction Prediction and a New Perspective Derived from Image Classification.” *Briefings in Bioinformatics* 22(4): bbaa318. https://doi.org/10.1093/bib/bbaa318

7. Montemurro, Alessandro, Viktoria Schuster, Helle Rus Povlsen, Amalie Kai Bentzen, Vanessa Jurtz, William D. Chronister, Austin Crinklaw, et al. 2021. “NetTCR-2.0 Enables Accurate Prediction of TCR-Peptide Binding by Using Paired TCRα and β Sequence Data.” *Communications Biology* 4(1): 1060. https://doi.org/10.1038/s42003-021-02610-3

8. Tickotsky, Nili, Tal Sagiv, Jaime Prilusky, Eric Shifrut, and Nir Friedman. 2017. “McPAS-TCR: A Manually Curated Catalogue of Pathology-Associated T Cell Receptor Sequences.” edited by J. Wren. *Bioinformatics* 33(18): 2924–29. https://doi.org/10.1093/bioinformatics/btx286

9. Springer, Ido, Nili Tickotsky, and Yoram Louzoun. 2021. “Contribution of T Cell Receptor Alpha and Beta CDR3, MHC Typing, V and J Genes to Peptide Binding Prediction.” *Frontiers in Immunology* 12: 664514. https://doi.org/10.3389/fimmu.2021.664514

10. Gao, Yicheng, Yuli Gao, Yuxiao Fan, Chengyu Zhu, Zhiting Wei, Chi Zhou, Guohui Chuai, et al. 2023. “Pan-Peptide Meta Learning for T-Cell Receptor–Antigen Binding Recognition.” *Nature Machine Intelligence*. https://doi.org/10.1038/s42256-023-00619-3

11. Bagaev, Dmitry V., Renske M. A. Vroomans, Jerome Samir, Ulrik Stervbo, Cristina Rius, Garry Dolton, Alexander Greenshields-Watson, et al. 2020. “VDJdb in 2019: Database Extension, New Analysis Infrastructure and a T-Cell Receptor Motif Compendium.” *Nucleic Acids Research* 48(D1): D1057–62. https://doi.org/10.1093/nar/gkz874

12. Dhanda, Sandeep Kumar, Swapnil Mahajan, Sinu Paul, Zhen Yan, Haeuk Kim, Martin Closter Jespersen, Vanessa Jurtz, et al. 2019. “IEDB-AR: Immune Epitope Database—Analysis Resource in 2019.” *Nucleic Acids Research* 47(W1): W502–6. https://doi.org/10.1093/nar/gkz452

13. Zhang, Wei, Longlong Wang, Ke Liu, Xiaofeng Wei, Kai Yang, Wensi Du, Shiyu Wang, et al. 2019. “PIRD: Pan Immune Repertoire Database.” edited by J. Kelso. *Bioinformatics* btz614. https://doi.org/10.1093/bioinformatics/btz614

14. Gowthaman, Ragul, and Brian G. Pierce. 2019. “TCR3d: The T Cell Receptor Structural Repertoire Database.” edited by J. Wren. *Bioinformatics* 35(24): 5323–25. https://doi.org/10.1093/bioinformatics/btz517

15. Lu, Manman, Linfeng Xu, Xingxing Jian, Xiaoxiu Tan, Jingjing Zhao, Zhenhao Liu, Yu Zhang, et al. 2022. “dbPepNeo2.0: A Database for Human Tumor Neoantigen Peptides From Mass Spectrometry and TCR Recognition.” *Frontiers in Immunology* 13: 855976. https://doi.org/10.3389/fimmu.2022.855976

16. Li, Guangyuan, Balaji Iyer, VB Surya Prasath, Yizhao Ni, and Nathan Salomonis. 2021. “DeepImmuno: Deep Learning-Empowered Prediction and Generation of Immunogenic Peptides for T-Cell Immunity.” *Briefings in Bioinformatics* 22(6): bbab160.

17. Lin, Zeming, Halil Akin, Roshan Rao, Brian Hie, Zhongkai Zhu, Wenting Lu, Nikita Smetanin, et al. 2023. “Evolutionary-Scale Prediction of Atomic-Level Protein Structure with a Language Model.” *Science* 379(6637): 1123–30. https://doi.org/10.1126/science.ade2574

18. Zheng, Chunhong, Joseph N. Fass, Yi-Ping Shih, Andrew J. Gunderson, Nelson Sanjuan Silva, Huayu Huang, Brady M. Bernard, et al. 2022. “Transcriptomic Profiles of Neoantigen-Reactive T Cells in Human Gastrointestinal Cancers.” *Cancer Cell* 40(4): 410-423.e7. https://doi.org/10.1016/j.ccell.2022.03.005

19. Tirosh, Itay, Benjamin Izar, Sanjay M. Prakadan, Marc H. Wadsworth, Daniel Treacy, John J. Trombetta, Asaf Rotem, et al. 2016. “Dissecting the Multicellular Ecosystem of Metastatic Melanoma by Single-Cell RNA-Seq.” *Science* 352(6282): 189–96. https://doi.org/10.1126/science.aad0501

20. Bailey, Peter, David K. Chang, Katia Nones, Amber L. Johns, Ann-Marie Patch, Marie-Claude Gingras, David K. Miller, et al. 2016. “Genomic Analyses Identify Molecular Subtypes of Pancreatic Cancer.” *Nature* 531(7592): 47–52. https://doi.org/10.1038/nature16965

## SUPPLEMENTARY FIGURE


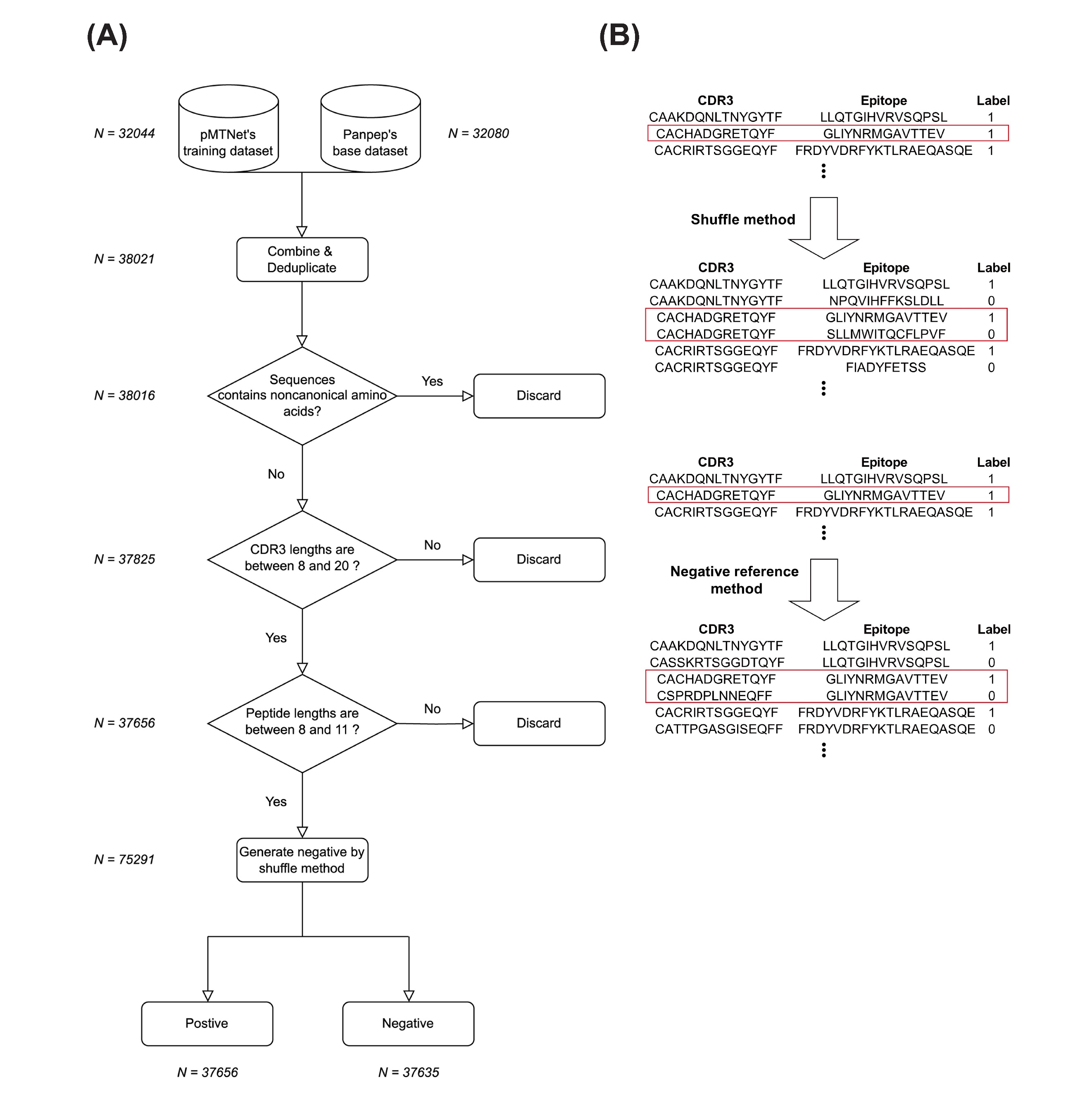


**Figure S1. Data processing workflow.** (A) Data processing for the CD8 training dataset as an example. (B) Shuffle method and negative reference method to generate negative data.


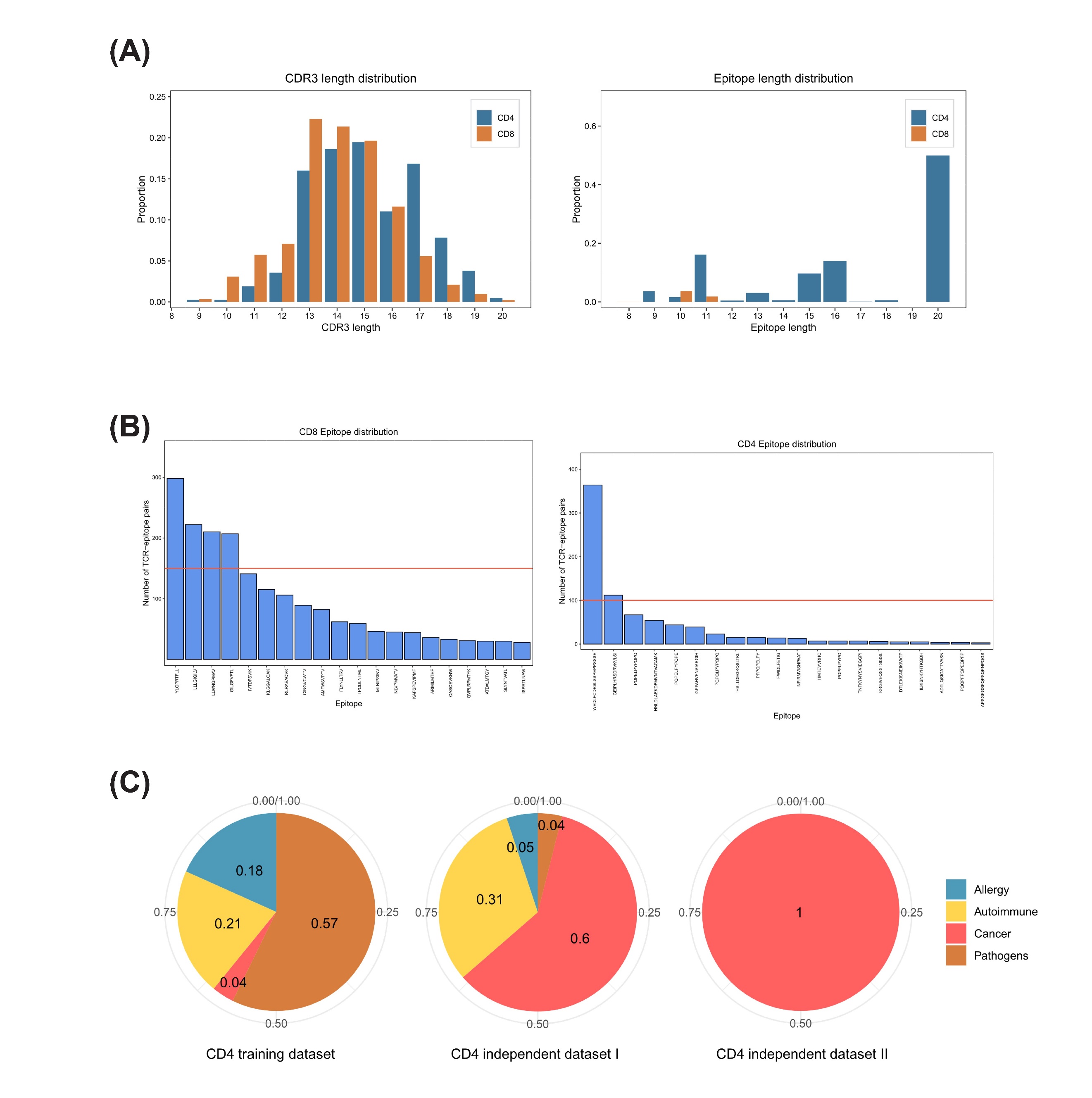


**Figure S2. Distribution of independent validation data for CD8 and CD4 models.** (A) Distribution of CDR3β (left) and epitope (right) lengths in both CD4 and CD8 independent datasets. (B) Number distribution of epitope pairs in both CD4 and CD8 independent datasets. (C) Distribution of epitope sources in CD4 datasets.


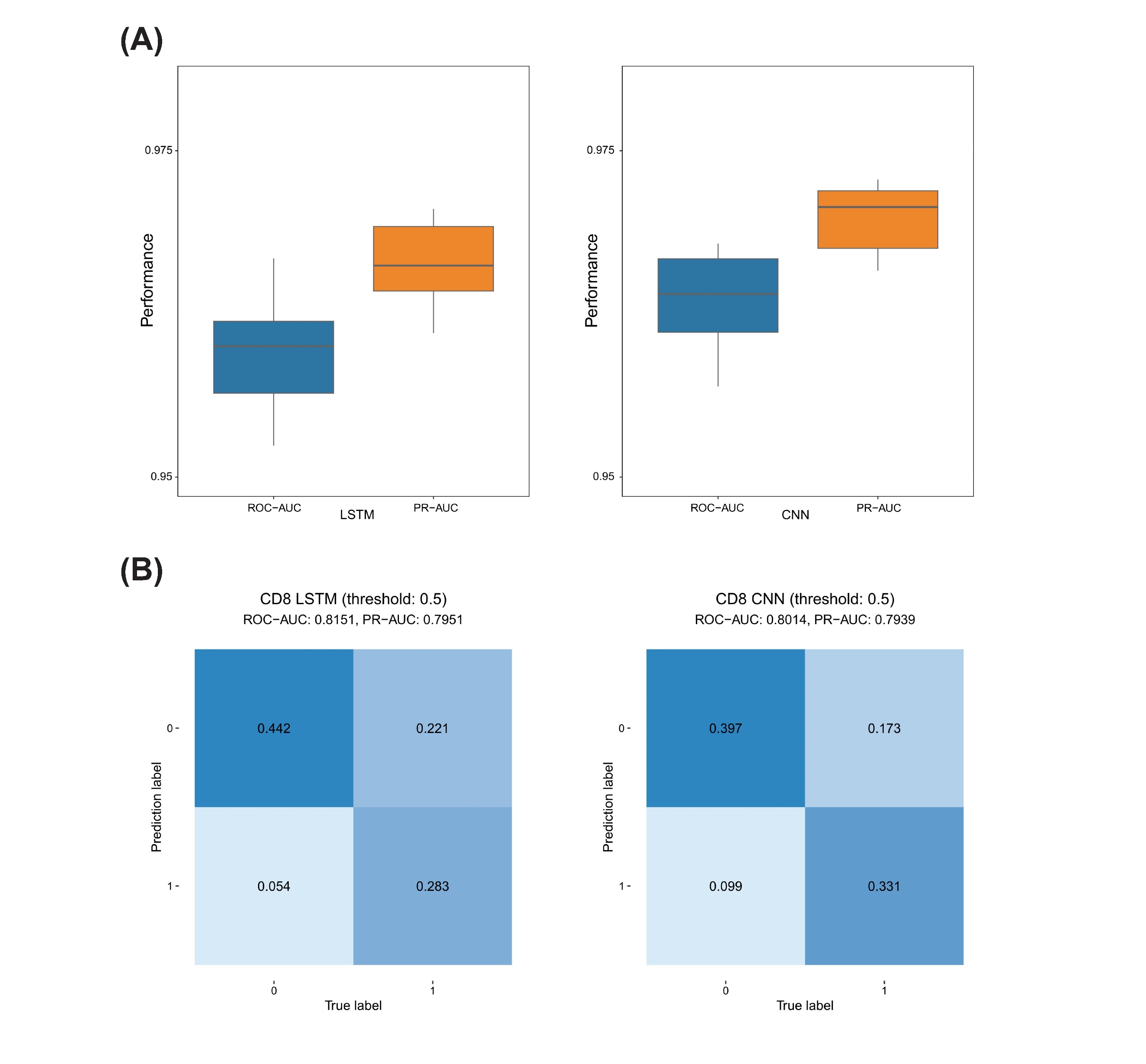


**Figure S3. The trained CD8 models demonstrate good generalization ability.** (A) 10-fold cross-validation of CD8 LSTM model (left) and CD8 CNN model (right) on the CD8 training dataset. (B) Confusion matrices for the CD8 LSTM model and CD8 CNN model on the CD8 independent validation dataset, with both thresholds set at 0.5.


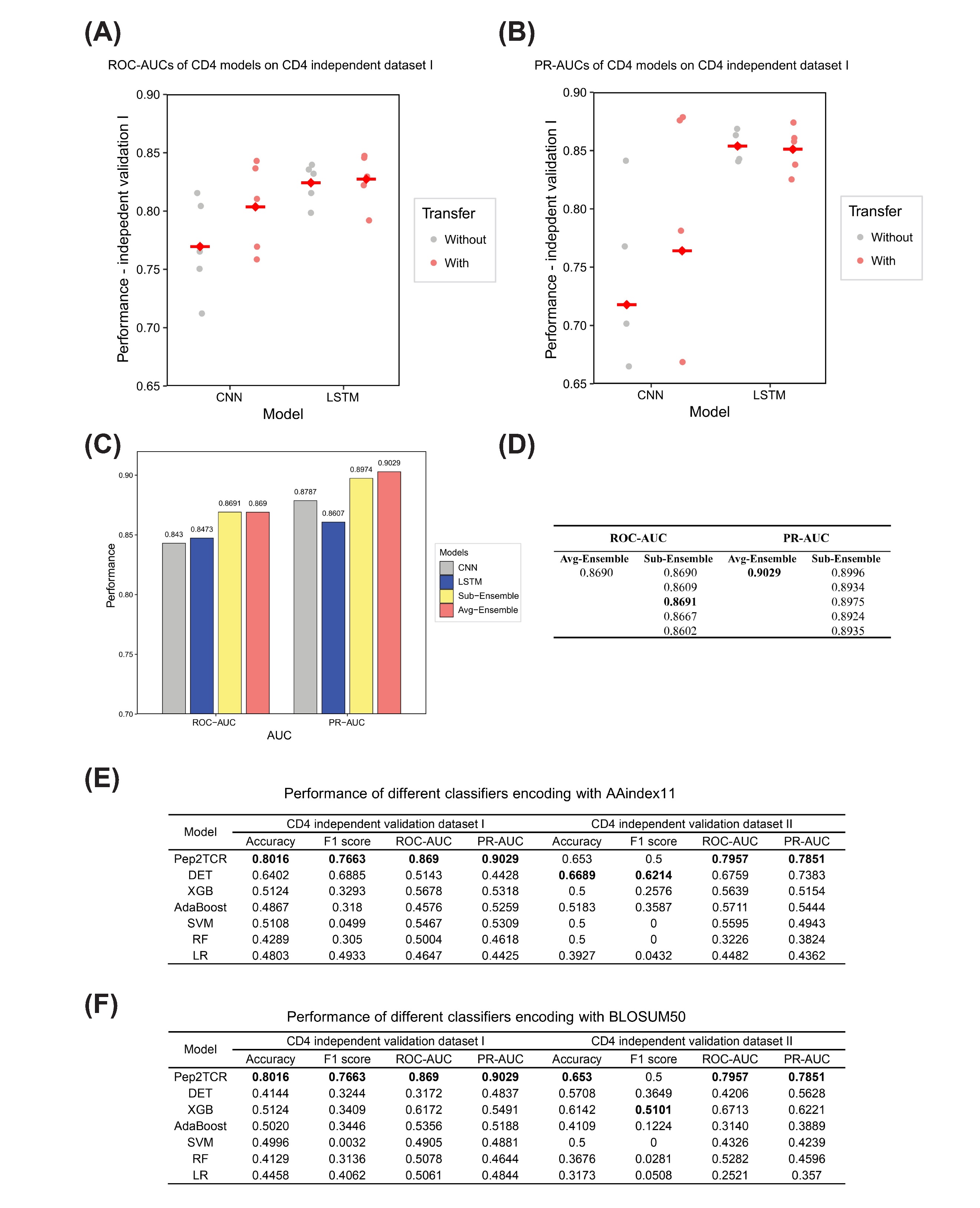


**Figure S4. Transfer learning and ensemble approach enhance the performance of TCR specificity prediction models.** (A-B) ROC-AUCs and PR-AUCs of 5-fold cross-validation of the CD4 models with and without transfer learning on the CD4 independent validation dataset I. (C) ROC-AUCs and PR-AUCs of CD4 LSTM, CD4 CNN, Avg-Ensemble and Sub-Ensemble models on the CD4 independent validation dataset I (chose the best CD4 LSTM and CD4 CNN models for comparison). (D) A table showed 5-fold cross-validation of the Avg-Ensemble model and Sub-Ensemble model on the CD4 independent validation dataset I. The performance of Pep2TCR and different classifiers encoding with AAindex11 (E) or BLOSUM50 (F) on the CD4 independent validation dataset I and II. Abbreviations: DET, decision tree; XGB, extreme gradient boosting; SVM, support vector machine; RF, random forest; LR, logistic regression.


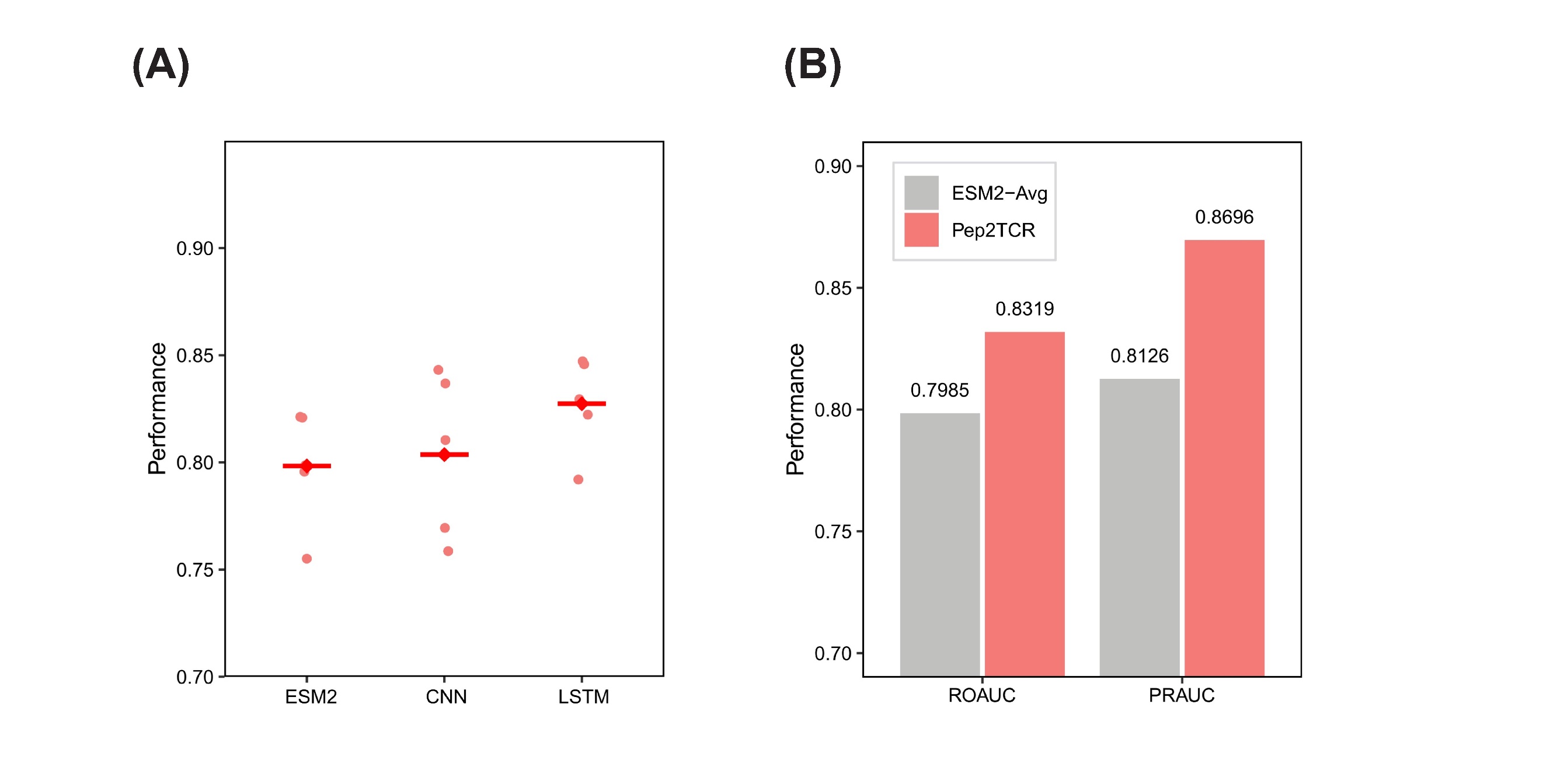


**Figure S5. Pep2TCR outperforms ESM2-based model.** (A) ROC-AUCs of transferred CD4 LSTM, CD4 CNN and ESM2-based model on the CD4 independent validation dataset I. (B) ROC-AUCs and PR-AUCs of Pep2TCR and ESM2-Avg ensemble model on the combined independent CD4 validation dataset.


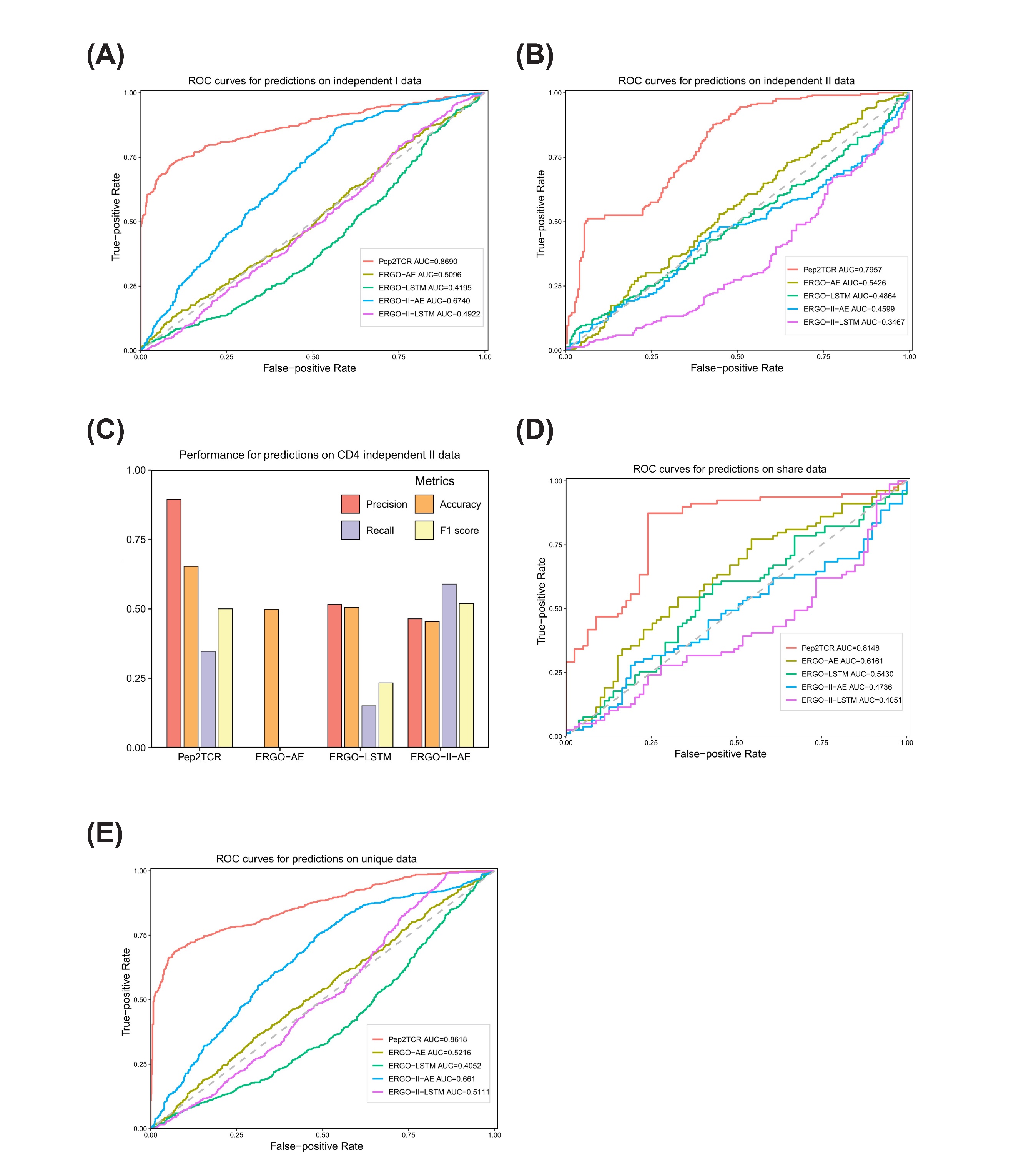


**Figure S6. Pep2TCR surpasses existing tools in CD4 TCR specificity prediction.** (A and B) ROC curves of Pep2TCR, ERGO and ERGOII on the CD4 independent validation dataset I (left) and II (right), respectively. (C) Precision, recall, f1 score and accuracy of Pep2TCR, ERGO and ERGOII on the CD4 independent validation dataset II. (D and E) The CD4 independent datasets I and II were divided as a share peptide dataset and a unique peptide dataset. The ROC curves of Pep2TCR, ERGO and ERGOII on the share dataset (left) and unique datasets (right) were demonstrated.


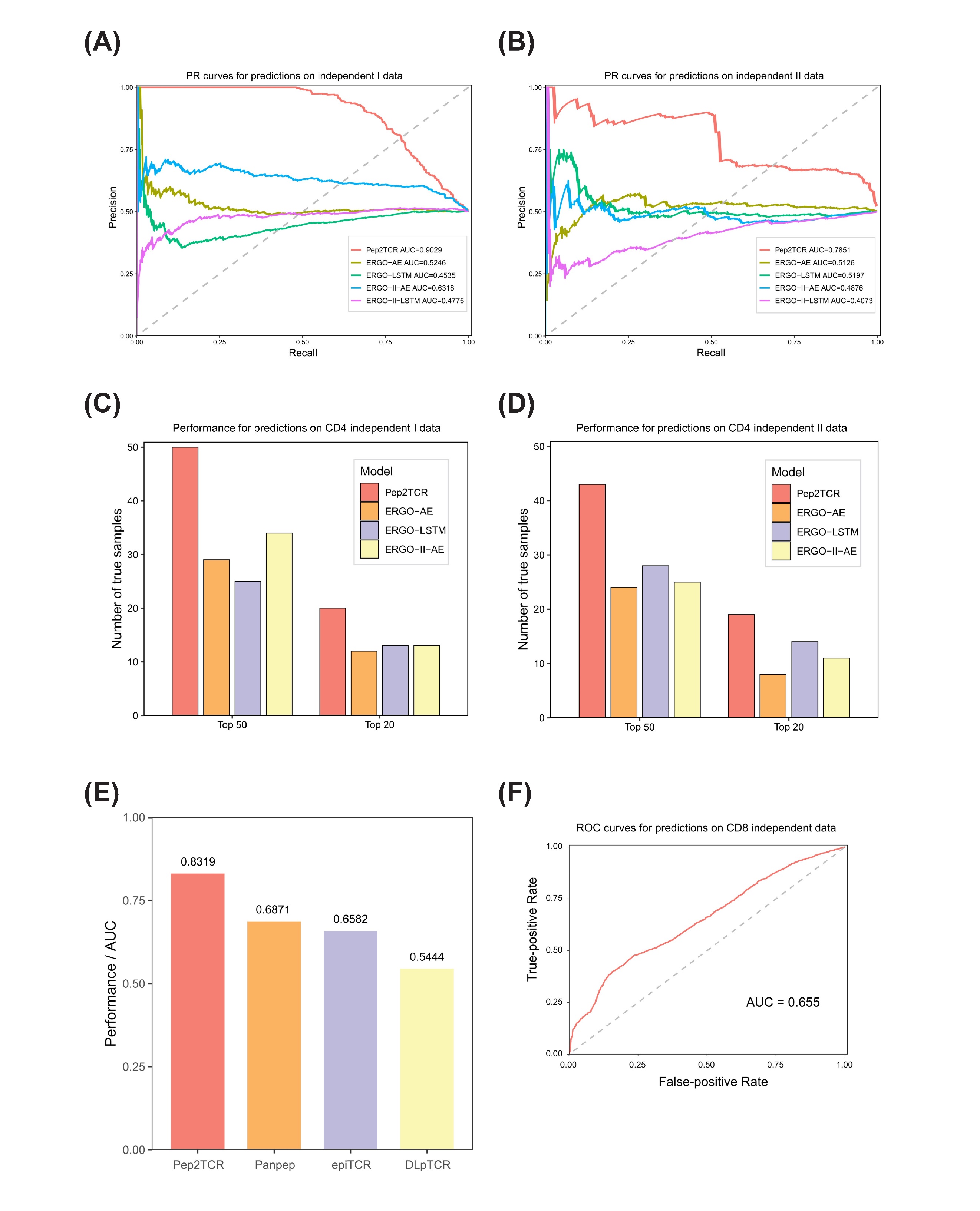


**Figure S7. Pep2TCR outperforms existing tools in CD4 TCR specificity prediction.** (A and B) PR curves of Pep2TCR, ERGO and ERGOII on the CD4 independent validation dataset I (left) and II (right), respectively. (C and D) Top50 and top20 values of Pep2TCR, ERGO and ERGOII on the CD4 independent validation dataset I (left) and II (right), respectively. (E) ROC-AUC values of Pep2TCR and major CD8 TCR specificity prediction tools on the combined independent CD4 validation dataset. (F) ROC-AUC value of Pep2TCR on the independent CD8 validation dataset.


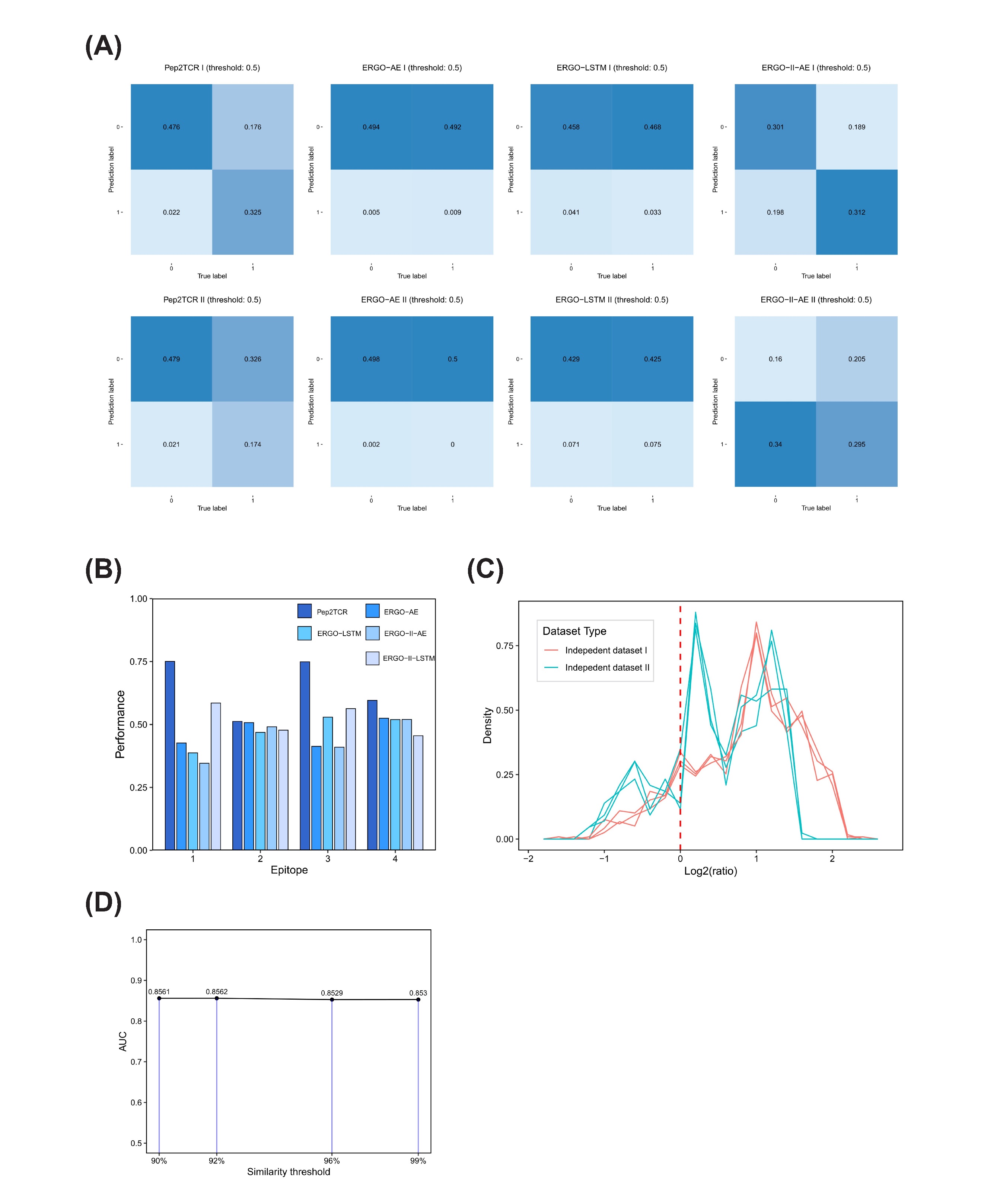


**Figure S8. Validating the performance of Pep2TCR.** (A) Confusion matrices for the Pep2TCR, ERGO and ERGOII on the CD4 independent validation dataset I (up) and II (down), with both thresholds set at 0.5. (B) ROC-AUCs of Pep2TCR and ERGO on four most common epitopes from the CD4 independent validation dataset. The four common epitopes are: 1. WEDLFCDESLSSPEPPSSSE, 2. GEIPLHRSDRVKVLSI, 3. PQPELPYPQPQ, 4. HNLDLAEKDFMVNTVAGAMK. (C) Pep2TCR can distinguish the different peptides corresponding to same CDR3s on the CD4 independent validation dataset I (red) and II (blue). The results were replicated three times. Given two peptides and a same CDR3 as an example (one of them is a positive sample, and the other is negative sample), $ratio= \frac{True preds}{False preds}$, $True preds$ represents the Pep2TCR prediction of the positive sample, $False preds$ is the opposite. (D) Overall AUCs assessed on the CD4 independent datasets at various similarity thresholds.

**
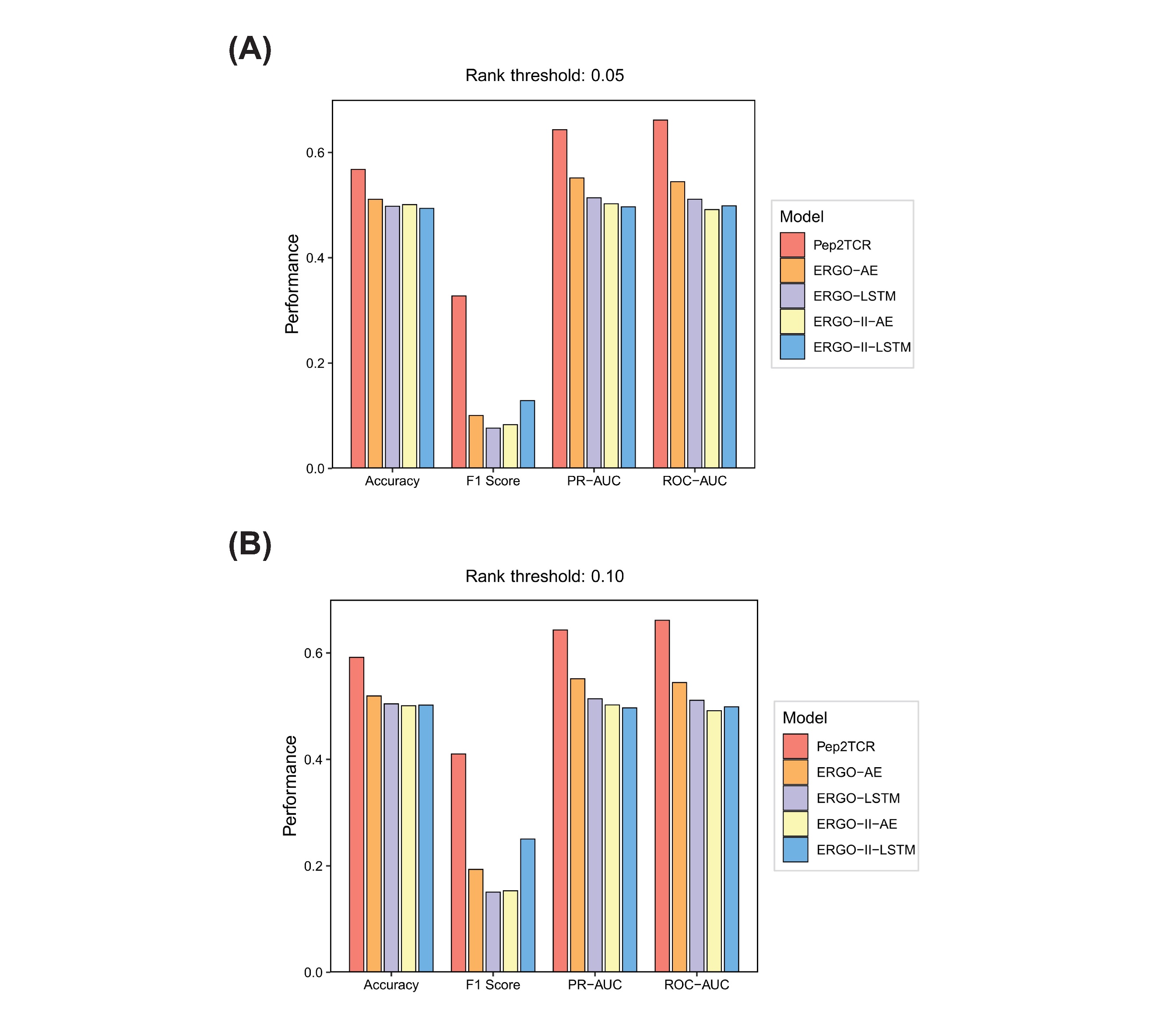
**

**Figure S9. Validation of Pep2TCR in the context of binding rank.** Comparing the performance of Pep2TCR and ERGO under the binding rank values. Thresholds are 0.05 (A) and 0.10 (B).


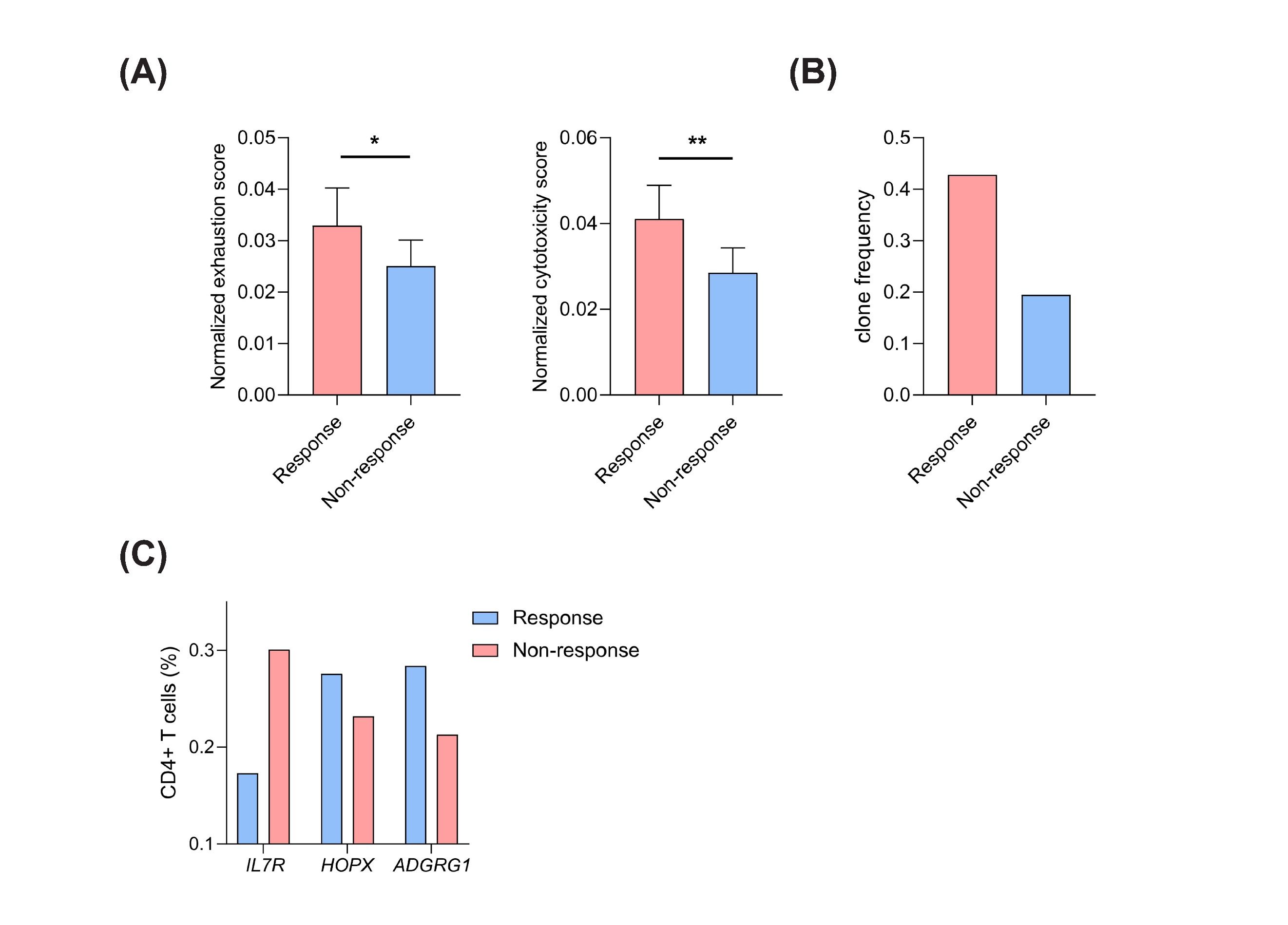


**Figure S10. Application of Pep2TCR in recognizing the signatures of neoantigen-reactive CD4^+^ T cell.** (A) Exhaustion scores and cytotoxicity scores of the neoantigen responsive group and non-responsive group (mean ± std). Here, max-min normalization was used to normalize both scores. The P values were calculated by one-sided t test. *****P value < 0.05, ******P value < 0.01. (B) Frequency of CD4^+^ T cells with cloned state in the response group and non-response group. (C) The ratio of CD4^+^ T cells with high levels of *IL7R*, *HOPX* or *ADGRG1* expression (exceeding the 0.75 percentile indicates a high level of expression) between the response group and non-response group.
